# Impact of the COVID-19 pandemic on prevalence of highly resistant microorganisms in hospitalised patients in the Netherlands, March 2020 to August 2022

**DOI:** 10.2807/1560-7917.ES.2023.28.50.2300152

**Published:** 2023-12-14

**Authors:** Wieke Altorf-van der Kuil, Cornelia CH Wielders, Romy D Zwittink, Sabine C de Greeff, Dave A Dongelmans, Ed J Kuijper, Daan W Notermans, Annelot F Schoffelen, on behalf of the study collaborators ISIS-AR study group

**Affiliations:** 1 Centre for Infectious Disease Control (CIb), National Institute for Public Health and the Environment (RIVM), Bilthoven, the Netherlands; 2 National Intensive Care Evaluation (NICE) Foundation, Amsterdam, the Netherlands; 3 Amsterdam University Medical Centers, Department of Medical Microbiology and Infection Prevention, Amsterdam, the Netherlands; 4 Amsterdam University Medical Centers location University of Amsterdam, Department of Intensive Care Medicine, Amsterdam, the Netherlands; 5 Members of the ISIS-AR study group are listed under acknowledgements

**Keywords:** Coronavirus disease (COVID-19), highly resistant microorganisms, antimicrobial resistance, multidrug resistance, surveillance

## Abstract

**Background:**

The COVID-19 pandemic resulted in adaptation in infection control measures, increased patient transfer, high occupancy of intensive cares, downscaling of non-urgent medical procedures and decreased travelling.

**Aim:**

To gain insight in the influence of these changes on antimicrobial resistance (AMR) prevalence in the Netherlands, a country with a low AMR prevalence, we estimated changes in demographics and prevalence of six highly resistant microorganisms (HRMO) in hospitalised patients in the Netherlands during COVID-19 waves (March–June 2020, October 2020–June 2021, October 2021–May 2022 and June–August 2022) and interwaves (July–September 2020 and July–September 2021) compared with pre-COVID-19 (March 2019–February 2020).

**Methods:**

We investigated data on routine bacteriology cultures of hospitalised patients, obtained from 37 clinical microbiological laboratories participating in the national AMR surveillance. Demographic characteristics and HRMO prevalence were calculated as proportions and rates per 10,000 hospital admissions.

**Results:**

Although no significant persistent changes in HRMO prevalence were detected, some relevant non-significant patterns were recognised in intensive care units. Compared with pre-COVID-19 we found a tendency towards higher prevalence of meticillin-resistant *Staphylococcus aureus* during waves and lower prevalence of multidrug-resistant *Pseudomonas aeruginosa* during interwaves. Additionally, during the first three waves, we observed significantly higher proportions and rates of cultures with *Enterococcus faecium* (pooled 10% vs 6% and 240 vs 120 per 10,000 admissions) and coagulase-negative Staphylococci (pooled 21% vs 14% and 500 vs 252 per 10,000 admissions) compared with pre-COVID-19.

**Conclusion:**

We observed no substantial changes in HRMO prevalence in hospitalised patients during the COVID-19 pandemic.

**Key public health message**

**What did you want to address in this study?**

If bacteria become resistant to antimicrobials, bacterial infections may become more difficult to treat. During the COVID-19-pandemic, the occurrence of such infections could have changed because of several factors, like more intensive infection control measures in hospitals, more patient transfers between hospitals and a reduction in traveling. We investigated whether the number of infections by the most important resistant bacteria changed during the COVID-19 pandemic.

**What have we learnt from this study?**

We did not observe any large and lasting changes in the occurrence of the six most important highly resistant bacteria in the Netherlands between March 2020 and August 2022 compared with the period before the COVID-19 pandemic (March 2019 to February 2020).

**What are the implications of your findings for public health?**

Our results suggest that the COVID-19 pandemic had no large short-term impact on the occurrence of the six most important highly resistant bacteria in the Netherlands. However, as antimicrobial resistance in the Netherlands may be influenced by other factors such as import from abroad it is important to continue monitoring antimicrobial resistance.

## Introduction

Already from the start of the COVID-19 pandemic, concerns were widely expressed that antimicrobial resistance (AMR) could become a major threat in the post-pandemic era [1-3]. The COVID-19 pandemic, with many patients needing medical care, has caused high work pressure in nursing wards, emergency departments and intensive care units (ICU), with a particular focus on COVID-19, possibly compromising routine infection prevention measures, such as changing and draping of infusion lines and use of selective decontamination (SDD) [3-5]. Furthermore, accessibility of first line healthcare for non-COVID patients decreased, less urgent medical procedures were delayed, patient transfers between hospitals increased and, in some situations, infection control and antimicrobial stewardship were interrupted [1,3,5]. Additionally, COVID-19 patients often developed nosocomial or secondary infections because of prolonged hospitalisation and were more frequently subjected to empirical use of broad-spectrum antimicrobials, especially in the beginning of the pandemic when many uncertainties existed concerning the course and outcomes of the disease [1,3-8]. In a review covering 10 studies from different countries, the majority of COVID-19 patients received antimicrobials, even though, on average, bacterial coinfection upon admission was present in less than 3.5% of the patients [8]. Additionally, superinfections in patients with severe COVID-19 caused by highly resistant microorganisms (HRMO) have also been reported, like carbapenemase-producing Enterobacterales, meticillin-resistant *Staphylococcus aureus* (MRSA), carbapenem-resistant *Acinetobacter baumannii* and *Pseudomonas aeruginosa* and *Candida auris* [1,4,8].

On the other hand, fewer elective healthcare transfers across borders and more stringent hygienic measures in healthcare settings to prevent spread of COVID-19 may have decreased prevalence and spread of resistant pathogens and risk for healthcare associated infections, lowering the need for antimicrobial treatment [1,3-5]. Examples of such measures are increased use of appropriate personal protective equipment, improved hand hygiene, infection control precautions and surface cleaning, postponement of elective surgery and visitor screening [1,3,5-7,9,10]. In parallel, in the community, there have been COVID-19-related changes that presumably resulted in reduced transmission of pathogenic and/or resistant bacteria, such as social distancing, use of face masks, emphasis on hand hygiene and respiratory etiquette, mandatory lockdowns, international travel restrictions, orders to stay and work at home, and lower healthcare service utilisation [1,3,5,6,10].

There is a need for better understanding of the influence of the above-mentioned changes on AMR prevalence, including the effect in regions with low AMR rates, such as the Netherlands [2,3]. Most studies published thus far have been single centre studies, or multi-centre studies during only 2–6 months and involved small numbers of isolates or patients [11-19]. Larger, nationwide studies from different countries are required that compare AMR levels before and during the COVID-19 pandemic. In the Netherlands, there is a national surveillance system on AMR [20]. We used data from this system to investigate the effect of the COVID-19 pandemic on AMR in hospitalised patients in the Netherlands, by analysing demographic characteristics and prevalence of several HRMO during the (inter)waves, compared with a year pre-COVID-19.

## Methods

### Setting and data collection

Data were extracted from the Dutch Infectious Disease Surveillance System for Antimicrobial Resistance (ISIS-AR) [20], in which participating clinical microbiology laboratories (CML) provide data on microbial species and antimicrobial susceptibility testing (AST) results from all positive cultures from medical routine diagnostics for which AST was performed. Furthermore, for each isolate, some epidemiological data about the patient are collected, including year and month of birth, sex and type of healthcare setting and department where the sample was taken. For the current retrospective study, we included data on diagnostic (infection-related) samples of hospitalised patients from 37 of 47 Dutch CML serving hospitals for which data were available from March 2019 to August 2022, with high coverage over the country (in 73% of postcode areas at least one isolate of > 5% of inhabitants was included). More information can be seen in Supplementary Figure S1 (Supplement 1).

The number of hospital admissions per day (further referred to as admissions, only available for ICUs) was collected from the National Intensive Care Evaluation (NICE) registry [21], by COVID-19 status at admission as measured by an reverse transcription PCR (RT-PCR) of the respiratory secretions or consistency of a computerised tomography (CT) scan with COVID-19 (i.e. a COVID-19 Reporting and Data System (CO-RADS) score of ≥ 4 indicating a high suspicion on COVID-19 in combination with the absence of an alternative diagnosis).

### Definition of periods

**Figure 1:**
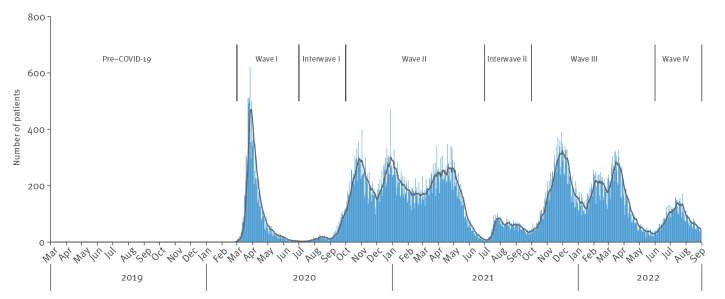
Timeline and daily number of newly admitted COVID-19 patients with a 7-day average, before and during the COVID-19 pandemic, the Netherlands, March 2019–September 2022 (n = 131,445)

We divided the COVID-19 pandemic until August 2022 into waves and interwaves, based on the number of COVID-19 hospital admissions in the Netherlands, as depicted in Figure 1. This resulted in four waves: wave I from March to June 2020, wave II from October 2020 to June 2021, wave III from October 2021 to May 2022 and wave IV from June to August 2022 and two interwaves: interwave i from July to September 2020 and interwave ii from July to September 2021. To compare results with data before COVID-19, we selected data from March 2019 to February 2020 as pre-COVID-19 period.

Based on data from the Dutch National Public Health Institute (RIVM), https://data.rivm.nl/covid-19/COVID-19_ziekenhuisopnames.csv).

### Definition of highly resistant microorganisms

We investigated prevalence of following six HRMO during the (inter)waves, compared with pre-COVID-19: extended spectrum β-lactamase (ESBL)-producing *Escherichia coli* and *Klebsiella pneumoniae*, carbapenemase-producing Enterobacterales (CPE) and *P. aeruginosa* (CPPA), multidrug-resistant *P. aeruginosa* (MDR-PA), meticillin-resistant *S. aureus* (MRSA) and vancomycin-resistant *Enterococcus faecium* (VRE). We interpreted all crude antimicrobial susceptibility test values according to the European Committee on Antimicrobial Susceptibility Testing (EUCAST) breakpoints version 11.0 [22]. Categorisation of *E. coli* and *K. pneumoniae* isolates as ESBL was based on results of ESBL confirmation tests, or, if data from these tests were lacking, on resistance to third generation cephalosporins (cefotaxime/ceftriaxone/ceftazidime), according to the most conservative breakpoint (> 1 mg/L). Categorisation of Enterobacterales as CPE and *P. aeruginosa* as CPPA was based on results of carbapenemase production confirmation tests (both the demonstration of phenotypical carbapenemase production and molecular detection of carbapenemase genes), or, if no data on confirmation tests were available, on phenotypical resistance to meropenem (according to the most conservative breakpoint of 2 mg/L) or imipenem. Isolates of *P. aeruginosa* were considered MDR-PA if resistant to more than three antimicrobial groups among fluoroquinolones (tobramycin), aminoglycosides (ciprofloxacin, levofloxacin), carbapenems (meropenem, imipenem), third generation cephalosporins (ceftazidime) and penicillins with β-lactamase inhibitor combinations (piperacillin-tazobactam). Categorisation of *S. aureus* isolates as MRSA was based on presence of *mecA* or *mecC* gene or pbp2 production, or, if data on these tests were lacking, on interpretation of cefoxitin susceptibility as determined by the CML, because raw testing values were often not available. If no information on a cefoxitin test was available, prevalence was based on laboratory interpretation of flucloxacillin/oxacillin susceptibility. Finally, *E. faecium* isolates were considered VRE based on presence of *vanA* or *vanB* gene, or, if these tests were lacking, on laboratory interpretation of amoxicillin/ampicillin and vancomycin susceptibility, with VRE being defined as resistant to amoxicillin/ampicillin and vancomycin.

### Statistical analyses

Data analysis was performed using SAS version 9.4 (SAS institute Inc., the United States). To analyse the number of patients and demographic patient characteristics, we selected the first sample per patient per (inter)wave, independent of isolated species determination. For each (inter)wave and type of department (ICU vs non-ICU), we calculated the number of patients with at least one positive bacterial culture in the dataset (further referred to as infected patients) and demographic characteristics of infected patients as percentages of all infected patients. For ICUs we additionally calculated the number of infected patients and the number of infected patients with at least one blood culture as rate per 10,000 admissions.

For analysis of the prevalence of Enterobacterales, *P. aeruginosa*, *Acinetobacter* spp., *E. faecium*, *S. aureus* and coagulase negative *Staphylococcus* spp. and HRMO, we selected the first isolate per species, per patient, per (inter)wave. Prevalence of these species, genus, or order was calculated as percentage of all selected positive cultures, stratified by department type, and additionally for ICUs as rate per 10,000 admissions in each (inter)wave. We calculated HRMO prevalence as the percentage of the total number of isolates of the corresponding species, genus, or order, stratified by department type, and additionally for ICUs as rate per 10,000 admissions in each (inter)wave. We calculated all percentages and rates with corresponding 95% Wald confidence intervals (95% CI). If the 95% CI of the estimate in one of the (inter)waves did not overlap with the 95% CI in the pre-COVID-19 period, the difference was considered statistically significant.

### Molecular analysis of meticillin-resistant *Staphylococcus aureus*

The results on MRSA prevalence during the (inter)waves required a deeper investigation on potential clustering. Therefore, we included molecular typing data of ICU MRSA isolates from the Dutch national MRSA surveillance database, where available. For all isolates in this database, multiple-locus variable number tandem repeat analysis (MLVA) data were available. Additionally, for part of the isolates multilocus sequence typing (MLST) data and whole genome multilocus sequence typing (wgMLST) data were available, as determined using next generation sequencing. A threshold of ≤ 16 allelic wgMLST differences was applied to determine genetic clusters.

## Results

### Patient demographics

**Figure 2:**
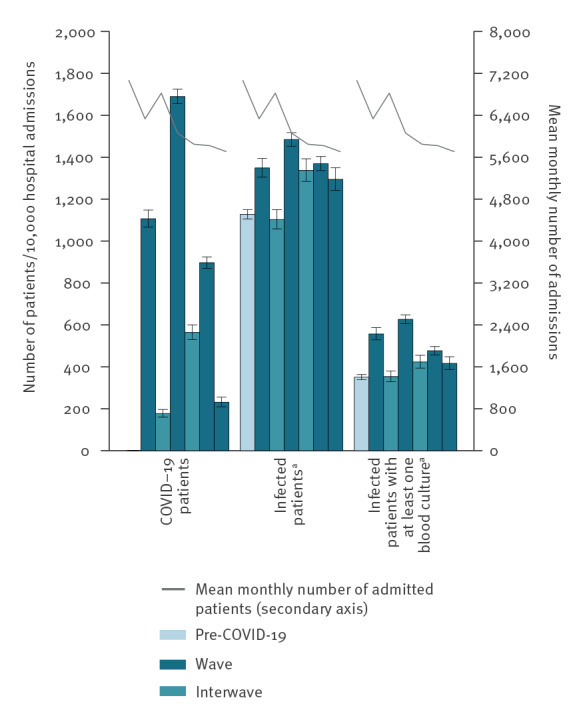
Rate per 10,000 new admissions of COVID-19 patients (n = 17,953), infected patients^a^ (n = 34,282) and infected patients^a ^(n = 12,008) with at least one positive blood culture, together with the mean monthly number of new admissions (n = 266,411), in ICU departments, by period, the Netherlands, March 2019–September 2022

In the Table, demographic characteristics of infected patients from ICU (n = 5,623) and non-ICU (n = 60,671) departments are shown by (inter)wave. Additionally, Figure 2 depicts for ICUs the rate of COVID-19 patients per (inter)wave, i.e. the number of COVID-19 patients per 10,000 admissions in each (interwave), the rate of infected patients, and, in more detail, the rate of infected patients with at least one positive blood culture. Additionally, the mean monthly number of admitted patients in ICUs is depicted as a line above each category. The main changes were found in ICUs, in which the percentage of COVID-19 admissions was between 9% (4,176/46,554) and 17% (9,217/54,551) during the first three waves, 2% (396/17,118) in wave IV and between 2% (363/20,473) and 6% (990/17,548) during interwaves. The mean monthly number of admissions was lower during the COVID-19 pandemic (ca 6,000) than pre-COVID-19 (7,070) and decreased over time. In contrast, compared with pre-COVID-19, the positive culture rate, as an approximation of the infection rate, was significantly 15–32% higher during the waves (range: 1,295 to 1,485/10,000 admissions during the waves vs 1,128/10,000 admissions pre-COVID-19, p value < 0.001) and 19% higher during interwave ii (1,337 vs 1,128, p value < 0.001). When focusing on the rate of positive blood cultures, we found a significant 19–79% higher rate during the waves (range: 417 to 627/10,000 admissions during the waves vs 351 pre-COVID-19, p value < 0.001) and a significant 21% higher rate in interwave ii (423 versus 351, p value < 0.001).

**Table:** Characteristics of infected patients (n = 5,623) in intensive care units and infected patients (n = 60,671) in other hospital units, before and during the COVID-19 pandemic, the Netherlands, March 2019–September 2022

Periods	Months	Admissions^a,^ ^c^	Infected patients^b^																			
			Total	Female	Male			Age category														
								0–19 years			20–39 years			40–59 years			60–79 years			≥ 80 years		
			n^c^	n^c^	n^c^	%^d^	95% CI	n^c^	%^d^	95% CI	n^c^	%^d^	95% CI	n^c^	%^d^	95% CI	n^c^	%^d^	95% CI	n^c^	%^d^	95% CI
**Intensive care units**																						
Pre-COVID-19	12	7,197	797	319	478	60	59–61	54	7	6–7	52	7	6–7	162	20	19–21	421	53	52–54	109	14	13–14
Wave I	4	6,333	855	290	564	66	64–68	54	6	5–7	49	6	5–7	215	25	24–27	482	56	55–58	56	7	6–7
Interwave i	3	6,824	753	288	465	62	60–64	70	9	8–11	58	8	7–9	164	22	20–23)	382	51	49–53	80	11	9–12
Wave II	9	6,061	900	299	601	67	66–68	61	7	6–7	44	5	4–5	215	24	23–25	522	58	57–59	58	6	6–7
Interwave ii	3	5,849	782	295	487	62	60–64	69	9	8–10	64	8	7–9	185	24	22–25	399	51	49–53	65	8	7–10
Wave III	8	5,819	797	293	504	63	62–64	66	8	8–9	61	8	7–8	187	23	22–24	426	53	52–55	57	7	7–8
Wave IV	3	5,706	739	287	451	61	59–63	78	11	9–12	58	8	7–9	174	24	22–25	358	48	46–51	71	10	8–11
**Other hospital units**																						
Pre-COVID-19	12	NA	8,687	4,518	4,169	48	48–48	568	7	6–7	905	10	10–11	1,340	15	15–16	3,718	43	42–43	2,156	25	25–25
Wave I	4		7,693	3,879	3,814	50	49–50	488	6	6–7	776	10	10–10	1,234	16	16–16	3,356	44	43–44	1,839	24	23–24
Interwave i	3		8,870	4,541	4,329	49	48–49	620	7	7–7	930	10	10–11	1,389	16	15–16	3,802	43	42–43	2,130	24	24–25
Wave II	9		8,091	4,179	3,913	48	48–49	492	6	6–7	828	10	10–10	1,277	16	16–16	3,533	44	43–44	1,961	24	24–25
Interwave ii	3		9,054	4,639	4,415	49	48–49	586	6	6–7	1,017	11	11–12	1,407	16	15–16	3,944	44	43–44	2,100	23	23–24
Wave III	8		8,844	4,569	4,274	48	48–49	576	7	6–7	918	10	10–11	1,315	15	15–15	3,825	43	43–44	2,211	25	25–25
Wave IV	3		9,432	4,791	4,641	49	49–50	634	7	6–7	1,006	11	10–11	1,460	15	15–16	4,107	44	43–44	2,224	24	23–24

CI: confidence interval; NA: not available.

Pre-COVID-19: March 2019–February 2020; wave I: March–June 2020; interwave i: July–September 2020; wave II: October 2020–June 2021; interwave ii: July–September 2021; wave III: October 2021–May 2022; wave IV: June–August 2022.

^a^ The number of admissions per day was collected from the National Intensive Care Evaluation (NICE) registry [21].

^b^ Patients with at least one positive bacterial culture in the dataset.

^c^ Mean monthly number.

^d^ Percentage of all infected patients within the department.

Data acquired from the Dutch national surveillance system for antimicrobial resistance (ISIS-AR).

ICU: intensive care unit.

Each group of bars shows from left to right the following periods: pre-COVID-19: March 2019–February 2020; wave I: March–June 2020; interwave i: July–September 2020; wave II: October 2020–June 2021; interwave ii: July–September 2021; wave III: October 2021–May 2022; wave IV: June–August 2022.

COVID-19 status was estimated based on a reverse transcription PCR of respiratory secretions of the patient or consistency of a computerised tomography scan with COVID-19 (i.e. a COVID-19 Reporting and Data System score of ≥ 4 indicating a high suspicion on COVID-19 in combination with the absence of an alternative diagnosis).

^a^ Patients with at least one positive bacterial culture in the dataset.

The main changes in infected patient demographics were observed in the ICUs: we found significantly higher proportions of infected male patients (ca 65% during the first three waves (564/855, 601/900 and 504/797 per month) vs 60% pre-COVID-19 (478/797)), infected patients aged 40–59 years (ca 24% in all waves (215/855, 215/900, 187/797 per month) and interwave ii (185/782) vs 20% pre-COVID-19 (162/797)) and patients aged 60–79 years (ca 57% in the first two waves (482/855 and 522/900 per month) vs 53% pre-COVID-19 (421/797)). The proportion of isolates from infected patients aged ≥ 80 years was significantly lower during all (inter)waves (6–11%, in total 62/824 per month)) compared with pre-COVID-19 (14%, 109/797). The proportion of infected patients aged 0–19 years was slightly lower during the first two waves, but significantly higher during the interwaves (9%, 70/753 and 69/782 per month) and wave III (8%, 66/797) and IV (11%, 78/739) compared with pre-COVID-19 (7%, 54/797).

### Bacterial cultures of infected patients

**Figure 3:**
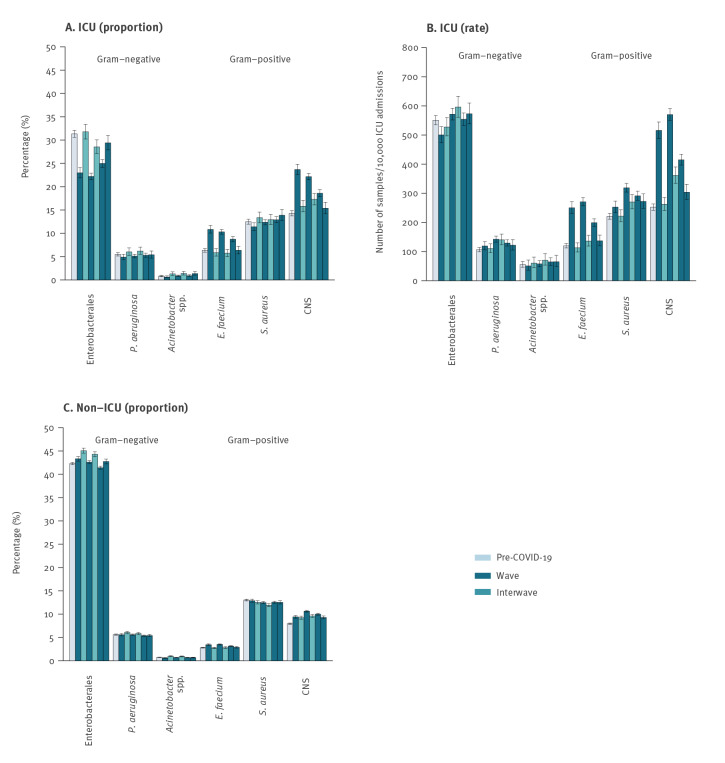
Proportions and rates of bacteria in samples from patients in intensive care units and other hospital units, the Netherlands, March 2019–September 2022

The main changes in species distribution were found in infected patients from the ICU. The proportion of species belonging to the order Enterobacterales was significantly lower during all waves compared with pre-COVID-19 (pooled 24% (7,946/33,222 positive cultures per month) vs 31% (4,674/14,921), p value < 0.03), although this was not reflected in the rates per 10,000 admissions (Figure 3). During the first three waves we found significantly higher prevalence of *E. faecium* (pooled 10% during the waves (2,948/29,886 positive cultures per month) vs 6% pre-COVID-19 (944/14,921, p value < 0.001) and pooled 240 per 10,000 admissions during the waves vs 120 pre-COVID-19, p value < 0.001). For CNS, the proportions were significantly higher in the first three waves (pooled 21%; 6,338/29,886) and in interwave ii (17%; 634/3,664), compared with pre-COVID-19 (14%, 178/12,43, p value < 0.001), whereas the rates were significantly higher during all waves and interwave ii (304–570/10,000 admissions during the waves and 361 during interwave ii vs 252 pre-COVID-19, p value < 0.001). Also, for *S. aureus* significantly higher rates were found (253–319/10,000 admissions during the waves and 270 during interwave ii, vs 221 pre-COVID-19, p value < 0.004), but when measured in proportions no significant differences were found in the (inter)waves vs pre-COVID-19.

CNS: coagulase negative *Staphylococci*; *E*.: *Enterococcus*; ICU: intensive care unit; *P*.: *Pseudomonas*; *S*.: *Staphylococcus*.

Each group of bars shows from left to right the following periods: pre-COVID-19: March 2019–February 2020; wave I: March–June 2020; interwave i: July–September 2020; wave II: October 2020–June 2021; interwave ii: July–September 2021; wave III: October 2021–May 2022; wave IV: June–August 2022.

### Prevalence of highly resistant microorganisms

**Figure 4:**
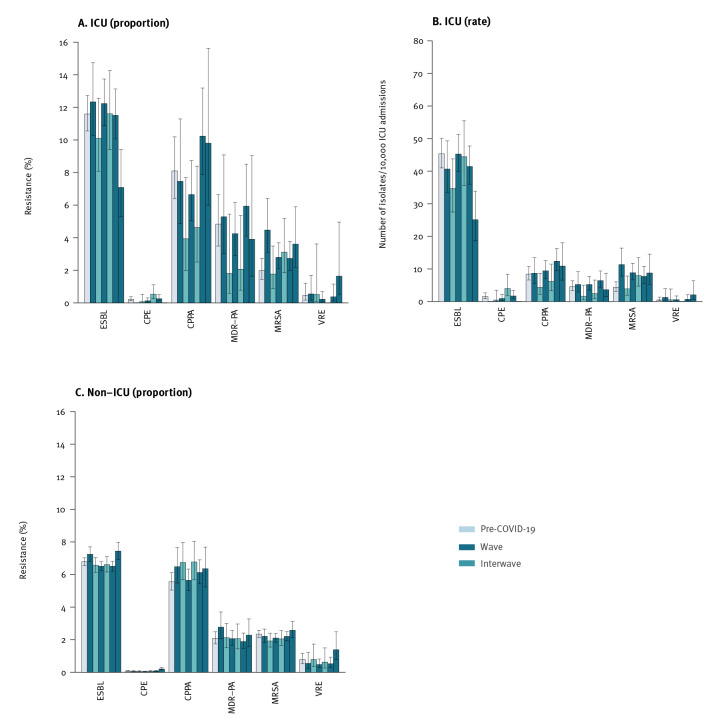
Proportion and rate of highly resistant microorganisms in intensive care units and other hospital units, the Netherlands, March 2019–September 2022

CPE: carbapenemase-producing Enterobacterales; CPPA:  carbapenemase-producing *Pseudomonas aeruginosa*; ESBL: extended spectrum β -lactamase-producing *Escherichia coli* and *Klebsiella pneumoniae*; ICU: intensive care unit; MDR-PA: multidrug-resistant *Pseudomonas aeruginosa*; MRSA: meticillin resistant *Staphylococcus aureus*; VRE:  vancomycin-resistant *Enterococcus faecium*.

The prevalence of most HRMO for both non-ICU and ICU patients was not significantly different during the (inter)waves compared with pre-COVID-19 (Figure 4). However, some patterns could be recognised within ICU patients. The proportions and rates of MRSA were higher during the waves than pre-COVID-19 (3% (35/1,281) to 4% (28/626) vs 2% (37/1,862), and 8–11 vs 4/10,000 admissions), with only the difference between wave I and pre-COVID-19 being significant for both outcomes (p value < 0.001). Both MRSA outcome measures decreased to pre-COVID-19 levels during interwave i (2% (8/453) and 4/10,000 admissions) but remained above pre-COVID-19 levels during interwave ii (3% (14/450) and 8/10,000 admissions, non-significant, p value > 0.05). From the national MRSA surveillance database, we could link MLVA results to 124 (67%) and MLST results to 42 (23%) of 185 MRSA isolates from the ICU. The isolates were classified into 83 different MLVA types and 18 sequence types. More information can be seen in Supplementary Table S1 (Supplement 2) and Figure S2 (Supplement 3). Two isolates, sampled in wave I, belonged to the same cluster. The 24 MRSA isolates from wave I were classified into 20 MLVA types and 11 sequence types. No apparent differences in MLVA types and sequence types were observed between (inter)waves and the pre-COVID-19 period.

Each group of bars shows from left to right the following periods: pre-COVID-19: March 2019–February 2020; wave I: March–June 2020; interwave i: July–September 2020; wave II: October 2020–June 2021; interwave ii: July–September 2021; wave III: October 2021–May 2022; wave IV: June–August 2022.

*Escherichia coli* and *Klebsiella pneumoniae* isolates were categorised as ESBL based on positivity of ESBL confirmation tests, or, if data from these tests were lacking, resistance to third generation cephalosporins (cefotaxime/ceftriaxone/ceftazidime), according to the most conservative breakpoint (> 1 mg/L).

Enterobacterales isolates were categorised as CPE and *Pseudomonas aeruginosa* isolates as CPPA on demonstration of phenotypical carbapenemase production and molecular detection of carbapenemase genes, or, if no data on confirmation tests were available, on phenotypical resistance to meropenem (according to the most conservative breakpoint (> 2 mg/L) or imipenem [22].

*Pseudomonas aeruginosa* isolates were categorised as MDR-PA if resistant to ≥ 3 antimicrobial groups among fluoroquinolones (tobramycin), aminoglycosides (ciprofloxacin, levofloxacin), carbapenems (meropenem, imipenem), third generation cephalosporins (ceftazidime) and penicillins with β-lactamase inhibitor combinations (piperacillin-tazobactam).

*Staphylococcus aureus* isolates were categorised as MRSA on presence of *mecA* or *mecC* gene or pbp2 production, or, if data on these tests were lacking, on laboratory interpretation of cefoxitin susceptibility as provided by the clinical microbiological laboratory, because raw testing values were often not available. If no information on a cefoxitin test was available, the categorisation was based on laboratory interpretation of flucloxacillin/oxacillin susceptibility.

*Enterococcus faecium* isolates were categorised as VRE on presence of *vanA* or *vanB* gene, or, if these tests were lacking, on laboratory S/I/R interpretation for amoxicillin/ampicillin and vancomycin, with VRE being defined as resistant to amoxicillin/ampicillin and vancomycin.

For CPPA and MDR-PA, we found a non-significant pattern with proportions and rates during the waves being similar to those pre-COVID-19, but lower during the interwaves (for CPPA: 4% (18/419) and 5 per 10,000 admissions in the interwaves vs 8% (65/803) and 9 per 10,000 admissions pre-COVID-19, p value > 0.05; for MDR-PA 2% (7/360) and 2 per 10,000 admissions vs 5% (35/724) and 5 per 10,000 admissions, p value > 0.09). During wave IV a significantly lower prevalence of 7% (43/607) and 4 per 10,000 admissions was found for ESBL compared with 12% (384/3,312) and 45 per 10,000 patients pre-COVID-19 (p value < 0.001), and a non-significantly higher prevalence of 2% (3/183) and 2 per 10,000 patients for VRE compared with 1% (4/876) and 1 per 10,000 admissions pre-COVID (p value > 0.06). No clear patterns could be distinguished for CPE prevalence.

## Discussion

In this retrospective study we used routine data from Dutch hospitalised patients and found changes in demographic data of infected patients, microorganism distribution and HRMO prevalence during the COVID-19 pandemic compared with pre-COVID-19, most prominently in ICUs. We found a higher rate of positive bacteriology cultures during the waves compared with pre-COVID-19. The isolates during the COVID-19 waves were obtained more often from male patients and patients aged 40–60 years and were more commonly Gram-positive bacteria. A pattern of higher proportions and rates of MRSA during the waves could be recognised, but only the difference between wave I and pre-COVID-19 was significant. Furthermore, we found a statistically non-significant pattern of lower proportions and rates of highly resistant *P. aeruginosa* during the interwaves. Findings in wave IV differed from those in the other waves with lower ESBL prevalence and statistically non-significant higher VRE prevalence.

Owing to comprehensiveness of the ISIS-AR database, we could enrol data on as much as on average 9,403 infected patients per month, leading to robust estimates and generally only limited impact of fluctuations in individual CML, caused by local clusters of HRMO or changes in laboratory protocols. Furthermore, due to the high geographical coverage over the Netherlands, the data can be considered representative for the whole country. Finally, because ISIS-AR is based on data from medical routine diagnostics, the estimates give a good reflection of the situation in the Dutch hospitals.

In addition to data from ISIS-AR, we used admission data for ICUs as provided by the NICE registry. This allowed us to calculate both proportions and rates of microorganisms and HRMOs, taking into account advantages of both approaches in interpretation of the data. Rates have the advantages that in the species distribution the measured prevalence of one species cannot be influenced by changes in the prevalence of the other species. Additionally, the HRMO prevalence is less influenced by policy changes influencing the ratio of resistant and susceptible bacteria, such as more stringent sampling policies focusing on population at risk for resistance or changing treatment strategies [23]. On the other hand, proportions are not sensitive to changes in the number of tested patients or infection rates. In the study of Amarsy et al. [24], a higher rate of bloodstream infections and infections with resistant bacteria was found in the COVID-19 pandemic compared with pre-COVID-19, whereas the proportion of resistant infections remained stable through time, which illustrates the importance to calculate both outcome measures.

A limitation of the current study is the lack of additional clinical information of patients, making it difficult to ascertain whether the observed changes could mainly be attributed to different biological characteristics and/or antimicrobial treatment of COVID-19 patients compared with the pre-COVID-19 patient population. Nevertheless, demographics of infected patients by (inter)wave showed shifts compared with pre-COVID-19 that were in line with published characteristics of COVID-19 patients [25]. Since there is no reason to assume that AMR differs by sex, we do not expect confounding influence on HRMO prevalence of the higher percentage of infected males. The higher proportion of isolates from relatively younger patients, however, may have lowered the observed HRMO prevalence. On the other hand, because COVID-19 patients stayed in average longer in ICUs compared with other patients, with a higher number of central venous catheter days and mechanical ventilation days, risk of nosocomial resistant and non-resistant infections was increased [8]. Still, other factors such as COVID-19-related infection prevention and control measures and behavioural aspects (such as social distancing and travel restrictions) will have affected occurrence and spread of resistant microorganisms. A second limitation is that no information on country of origin and country of potential infection or hospitalisation abroad were available in the surveillance system, making it impossible to infer whether patients were infected with an HRMO abroad.

The admission numbers at ICUs were lower during the waves and interwave ii compared with pre-COVID-19. Although such data were not available for this study, we hypothesise this was probably due to longer ICU stay per COVID-19 patient. The higher positive culture rate, as an approximation for infection rate, during the waves suggests that patients had bacterial infections more often than the pre-COVID-19 hospital population, probably due to more hospital-acquired infections caused by longer hospital stay [8,26] and more severe illness in non-COVID-19 patients being admitted due to strict triaging policies.

*Enterococcus faecium* and CNS, which both showed higher prevalence in ICUs during the waves, are important pathogens in venous catheter-related bloodstream infections (CRBSI) [27], which occurred relatively often in COVID-19 patients [28]. Nevertheless, the increase may also partly be explained by a higher number of blood samples taken due to the longer hospital stay of COVID-19 compared with the pre-COVID-19 population, herewith increasing the number of contaminated samples, incorrectly categorised as samples from infected patients [28]. The higher proportion of CNS that we found both in blood and in materials other than blood, may also partly have been caused by a higher contamination rate due to the combination of high work pressure and the necessity to work with personal protective equipment. *Staphylococcus aureus* is an important pathogen in both ventilator-associated pneumoniae (VAP) and CRBSI which could explain the higher rates in ICUs during the waves. The lower proportions of Enterobacterales during the waves may merely reflect higher prevalences of Gram-positive bacteria instead of a truly lower Enterobacterales prevalence. This hypothesis was confirmed by the fact that the rates did not show this pattern. All observed changes are in line with previous studies [16,18,29].

Conflicting results on AMR prevalence during the COVID-19 pandemic have been reported [9,10,13,15-17,24,29-31]. In European countries, however, within our study period mostly decreasing or stabilising trends were found [10,29,31]. In the Netherlands, pre-COVID-19 prevalence of HRMO was low (Figure 4), and most changes in HRMO prevalence were not significant. However, within ICUs a pattern could be recognised of higher MRSA prevalence during the waves, being significant in wave I. Molecular typing data of a subgroup of the MRSA isolates showed a diverse MRSA type distribution throughout COVID-19 (inter)waves, indicating that the observed increase of MRSA during wave I cannot evidently be attributed to increased transmission nor to a common source. In an Italian study on *S. aureus* VAP, De Pascale et al. found that COVID-19 patients were more likely to have late-onset VAP, with a larger proportion of MRSA in COVID-19 patients compared with non-COVID-19 patients [26]. However, the number of nosocomial infections with MRSA is expected to be low, due to the low baseline MRSA prevalence of 2% in Dutch hospitals. During wave I, the region in the Netherlands most affected by COVID-19 overlapped with the country’s main livestock area [32]. Therefore, we considered whether livestock farmers could have added more than usual to MRSA prevalence. However, because the higher MRSA prevalence was also found in infected patients from other regions in the Netherlands, we deemed this hypothesis unconvincing. A more likely explanation may be that a relatively higher proportion of the COVID-19 patients during the first part of wave I had a migration background and/or was infected abroad, before travel restrictions were established [33]. Some may have been colonised or infected with MRSA in a hospital there. Indeed, the higher prevalence of MRSA was mainly attributable to the first and second month of wave I, and returned to previous levels during interwave i. However, we could not find any explanation for the higher levels in interwave ii. Nevertheless, it should be emphasised that the difference between MRSA prevalence in interwave ii compared with pre-COVID-19 was not significant and may be a chance finding.

Although resistance levels in isolates of *P. aeruginosa* (CPPA and MDR-PA) during the waves remained similar to pre-COVID-19, we found a non-significant pattern of lower resistance proportions during interwaves. In a pre-COVID-19 study among two Dutch ICUs the main source for acquisition of VIM-carrying *P. aeruginosa*, was persistently contaminated environment (86%), but 14% of acquisitions were estimated to originate from patient-to-patient transfer [34]. Possibly, intensified hygienic measures during the COVID-19-pandemic may have prevented an increase in transmission from the environment to patients or between patients, resulting in lower levels during the interwaves.

During wave IV we found higher prevalence of VRE. However, this proved to be mainly attributable to a large outbreak in one hospital. Remarkably, ESBL prevalence in ICUs during wave IV was lower compared with pre-COVID-19 and other (inter)waves. During wave IV the COVID-19 admission rate to ICUs was low, and ICU population characteristics were different from other waves with higher proportion of younger infected patients (0–19 years) and lower proportion of patients 60–79 years. This might have influenced ESBL prevalence, but it is difficult to determine the cause of this finding.

## Conclusions

Using data from the Dutch national AMR surveillance system we found a shift in demographics of patients infected with HRMO that was in line with COVID-19 patient characteristics. Nevertheless, we found no substantial persistent changes in HRMO prevalence in hospitalised patients in the Netherlands during the COVID-19 pandemic, in spite of adaptation of infection control measures, increased patient transfer and decreased travelling abroad. AMR prevalence in the Netherlands is generally low, but still influenced by developments worldwide, as HRMO findings are regularly related to import from abroad. Therefore, it remains to be seen what the long-term impact of COVID-19 and its associated healthcare and societal changes on AMR prevalence in the Netherlands will be. Extra vigilance through continuous AMR surveillance is warranted.

## Supplementary Data

8782000 Supplementary Material 1

## Supplementary Data

81000 Supplementary Material 2

## Supplementary Data

540000 Supplementary Material 3
