# Supplementary material for: Impact of the COVID-19 pandemic on prevalence of highly resistant microorganisms in hospitalised patients in the Netherlands, March 2020 to August 2022
Source: Euro Surveill. 2023 Dec 14;28(50):2300152. doi: 10.2807/1560-7917.ES.2023.28.50.2300152 (PMC10831414; doi:10.2807/1560-7917.ES.2023.28.50.2300152)
Supplement: Supplementary Material 1 [file 23-00152_ALTORF-VANDERKUIL_Supplement1.pdf]

Supplementary figure S1. Smoothed geographical distribution of infected patients that were included in the analysis, based on the percentage of inhabitants for whom at least one isolate was included, by 4-digit postal code area and with regional cooperative network borders.

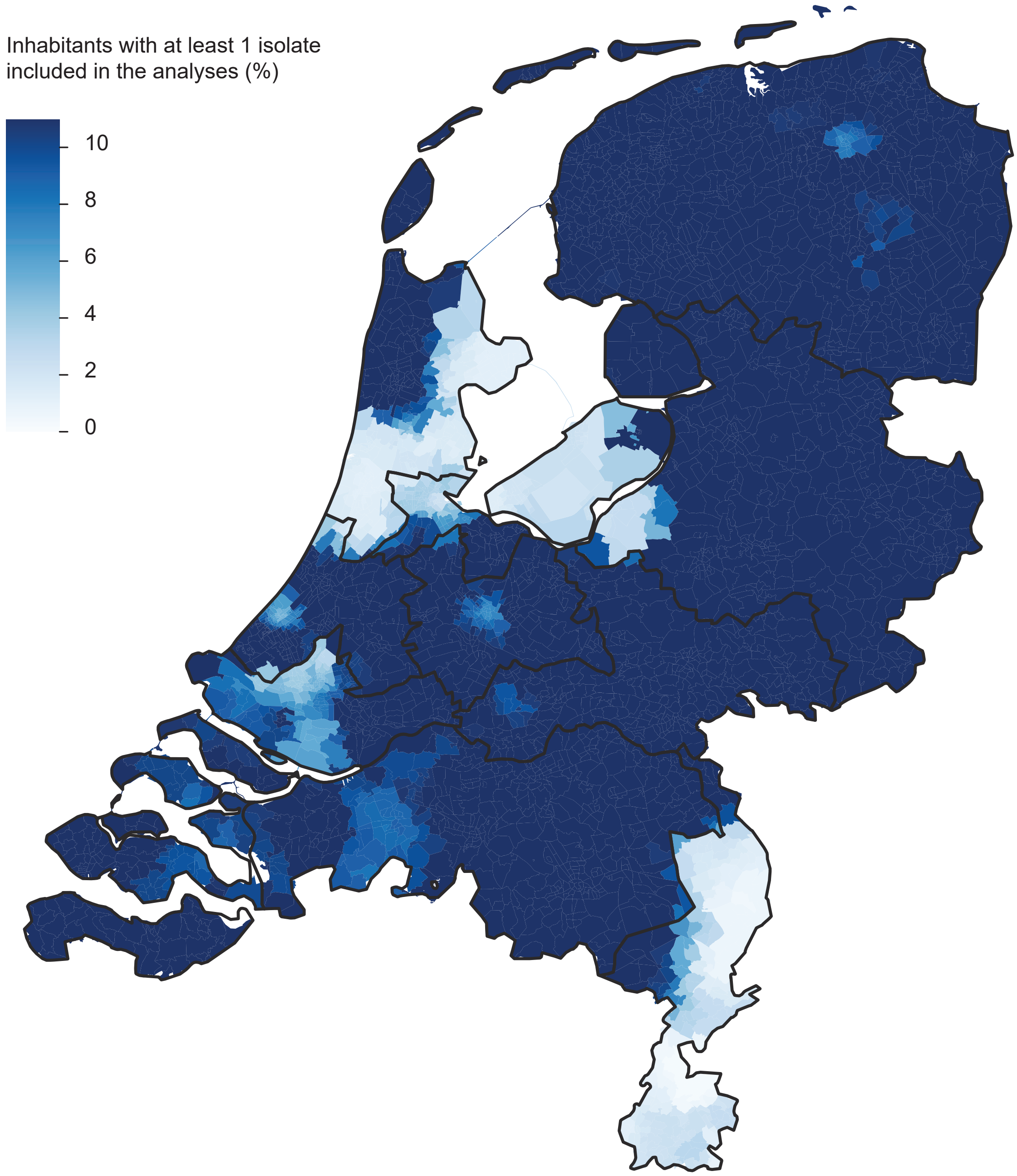

This supplementary material is hosted by Eurosurveillance as supporting information alongside the article 'Impact of the COVID-19 pandemic from march 2020 to August 2022 on prevalence of highly resistant microorganisms in hospitalised patients in the Netherlands', on behalf of the authors, who remain responsible for the accuracy and appropriateness of the content. The same standards for ethics, copyright, attributions and permissions as for the article apply. Supplements are not edited by Eurosurveillance and the journal is not responsible for the maintenance of any links or email addresses provided therein.
