# Supplementary material for: Impact of the COVID-19 pandemic on prevalence of highly resistant microorganisms in hospitalised patients in the Netherlands, March 2020 to August 2022
Source: Euro Surveill. 2023 Dec 14;28(50):2300152. doi: 10.2807/1560-7917.ES.2023.28.50.2300152 (PMC10831414; doi:10.2807/1560-7917.ES.2023.28.50.2300152)
Supplement: Supplementary Material 2 [file 23-00152_ALTORF-VANDERKUIL_Supplement2.pdf]

Supplementary table S1. MRSA type distribution throughout the study period as determined through MLVA typing (n=124, 67% of all included MRSA isolates) and NGS-based multi-locus sequence typing (n=42, 23% of all included MRSA isolates).

	MLVA		NGS	
	**Number of MRSA isolates**	**Number of different MLVA types**	**Number of MRSA isolates**	**Number of different sequence types**
Pre-COVID-19	12	10	2	2
Wave I	24	20	14	11
Interwave i	5	5	1	1
Wave II	37	32	16	9
Interwave II	10	9	1	1
Wave III	29	24	7	7
Wave IV	7	7	1	1
Total study period	124	83	42	18

This supplementary material is hosted by Eurosurveillance as supporting information alongside the article 'Impact of the COVID-19 pandemic from march 2020 to August 2022 on prevalence of highly resistant microorganisms in hospitalised patients in the Netherlands', on behalf of the authors, who remain responsible for the accuracy and appropriateness of the content. The same standards for ethics, copyright, attributions and permissions as for the article apply. Supplements are not edited by Eurosurveillance and the journal is not responsible for the maintenance of any links or email addresses provided therein.
