# Supplementary material for: Impact of the COVID-19 pandemic on prevalence of highly resistant microorganisms in hospitalised patients in the Netherlands, March 2020 to August 2022
Source: Euro Surveill. 2023 Dec 14;28(50):2300152. doi: 10.2807/1560-7917.ES.2023.28.50.2300152 (PMC10831414; doi:10.2807/1560-7917.ES.2023.28.50.2300152)
Supplement: Supplementary Material 3 [file 23-00152_ALTORF-VANDERKUIL_Supplement3.pdf]

Supplementary figure S2. Whole-genome MLST minimum spanning tree of 42 ICU MRSA isolates labeled according to COVID-19 (inter)wave (A) and multi-locus sequence type (B). Each circle symbolizes an MRSA isolate. Numbers indicate the number of allelic differences between isolates. The genetic cluster (zero allelic differences, 2 isolates) is depicted with a larger circle within a light-grey halo.

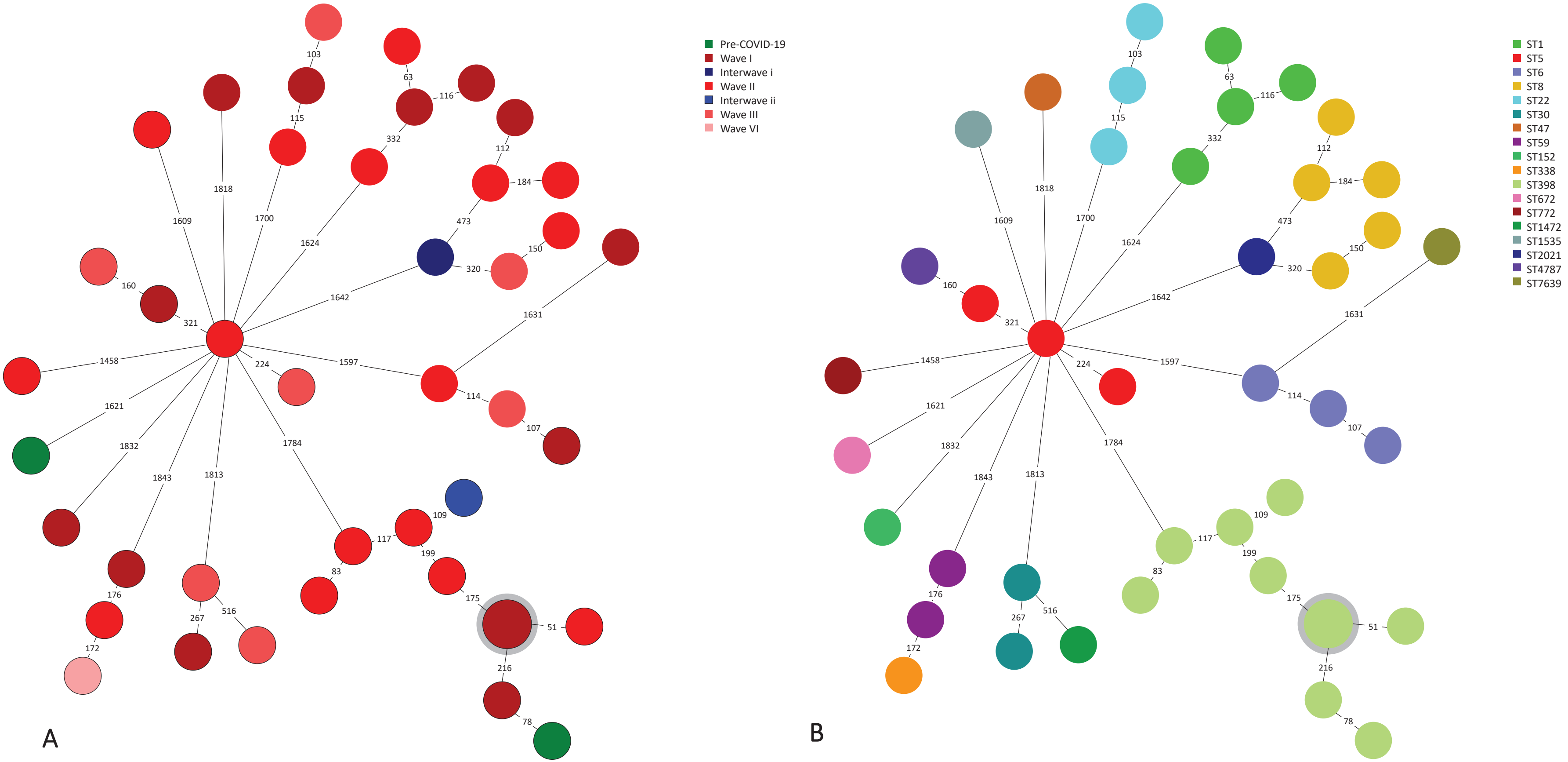

This supplementary material is hosted by Eurosurveillance as supporting information alongside the article ' Impact of the COVID-19 pandemic on prevalence of highly resistant microorganisms in hospitalised patients in the Netherlands, March 2020 to August 2022', on behalf of the authors, who remain responsible for the accuracy and appropriateness of the content. The same standards for ethics, copyright, attributions and permissions as for the article apply. Supplements are not edited by Eurosurveillance and the journal is not responsible for the maintenance of any links or email addresses provided therein.
